# Monoclonal Antibodies-Anchored Quantum Dots-Based Delivery Strategies for Glioblastoma Treatment: Challenges and Applications

**DOI:** 10.34172/apb.025.44026

**Published:** 2025-06-02

**Authors:** Dipak B. Bari, Chandrakantsing V. Pardeshi

**Affiliations:** R.C. Patel Institute of Pharmaceutical Education and Research, Industrial Pharmacy Laboratory, Department of Pharmaceutics, Shirpur 425405, Maharashtra, India

**Keywords:** Monoclonal antibody, Quantum dots, Glioblastoma, Conjugation, Cancer, Tumor

## Abstract

Treatment of glioblastoma multiforme (GBM) has been a great challenge before medical fraternity since last century owing to a median survival of less than 15 months, despite of intensive therapy. Neurosurgeries, intense chemotherapy, advanced radiotherapy, and targeted therapies have bought some extension to the life of GBM patients. Combination and targeted therapies could bring a concrete approach to tackle the complexities of GBM treatment. Monoclonal antibodies (mAbs) have already proved their potential, owing to their high affinity and target-specificity, as a promising cancer immunotherapy. In addition, the unique optical properties of quantum dots (QDs) make them an ideal choice of nanocarrier for delivering the chemotherapeutic agents across the blood-brain barrier (BBB) and blood-tumor barrier (BTB). Present review is a concise compilation of the investigations on mAbs conjugation on the QDs surface and their anticancer efficacy against GBM. The core purpose of this review is to discuss the major challenges in the current treatment of GBM and how the mAbs-conjugated QDs have enhanced the therapeutic efficacy in the targeted immunotherapy of GBM tumor. At the end of the article, authors have briefed about the current clinical status of mAbs in GBM treatment, which would urge the researchers to explore them in conjugation with the QDs-based delivery systems. Advancements in this strategy could further open the potential avenues in the future treatments of GBM.

## Introduction

 In healthy cells, a tightly controlled system acts to maintain the tissue homeostasis. Several genes *viz.* p53 (tumor suppressor), Bcl-2 (anti-apoptotic), and Bax (pro-apoptotic) are the crucial troupes who regulates the cell proliferation and apoptosis mechanisms. This ensures the appropriate growth and multiplication through signaling pathways when a cell should divide or undergo a programmed cell death whenever damaged.^1,2^ Due to genetic mutations, cancer cells are unable to respond to several signals that regulate cellular development and death.

 One of the most widely used cancer treatment techniques is chemotherapy. It uses cytotoxic drugs to destroy rapidly proliferating cells by interfering with the synthesis of DNA and replication of cells.^3^ Chemotherapy has been associated to harmful systemic side effects *viz.* nausea, vomiting, hair loss, exhaustion, and mouth sores because the drugs are nonselective and destroys rapidly proliferating cells without discrimination.^4^ Clinical therapies, particularly cancer therapy, have been profoundly altered by nanotechnology. By encapsulating the chemotherapeutics and releasing it at specific locations, nanoscale drug carriers can improve therapeutic effectiveness without increasing the dose and minimize the toxicity to healthy cells. Nanocarriers could increase the concentration of drug at the target site *via* target-specific delivery.^5,6^ Diverse nanocarriers *viz.* dendrimers, solid lipid nanoparticles, hydrogels, micelles, metal-organic frameworks, liposomes, and quantum dots (QDs), have been studied as delivery systems for antineoplastic chemotherapeutic drugs. QDs have been the most often utilized of these emerging nanocarriers in cancer therapeutic areas.^7,8^ The current pharmaceutical research is devoted to confirming the promise of several monoclonal antibodies (mAbs)-anchored QDs as therapeutic carriers in cancer, more specifically in glioblastoma multiforme (GBM), treatment.

###  Quantum dots 

**Figure 1:**
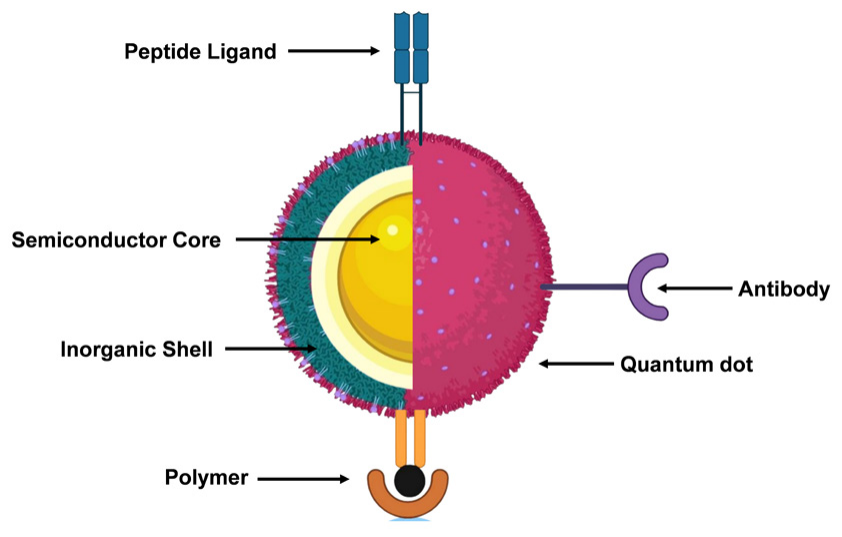
Structural presentation of the quantum dot with various bioactive ligands

 One of the extremely crucial components of nanomaterials-based drug delivery is QDs, which are semiconductors with a nanoscale dimension.^9^ Because of their low tissue absorption and decreased light dispersion, the near-infrared spectrum is ideal for biomedical imaging and drug delivery.^10,11^ The therapeutic potential of QDs with tunable optical characteristics has attracted the scientific community, in recent past, for their utilization in anticancer drug delivery.^12^ QDs have a greater likelihood of being used in biological applications because of their tiny size.^13^ It has been well known fact that QDs coated with biocompatible polymers, peptides, or antibodies (Figure 1) can deliver drugs to the target cells or tissues. They can also be used as bioimaging contrast materials for high-resolution imaging of biological processes and structures.^14^

 Scientists employ less-toxic biocompatible carbon nanodots to overrule the toxicity associated with the QDs synthesized from heavy metals.^15,16^ QDs find applications in biology,^17^ optoelectronics,^18^ electronics,^19^ and catalysis.^20^ They have intriguing pharmaceutical applications due to their distinct characteristics and variety of core materials utilized in their synthesis.^21^ Additional studies are being conducted to address the toxicity concerns and identify alternative synthesis approaches.^22^ By focusing on disease biomarkers, QDs can improve personalized treatments, increase the effectiveness of drug administration, and precision of bioimaging.^23,24^ QDs may revolutionize the GBM tumor diagnosis and treatment owing to its ultra-small size (~100 to 10 000 times) compared to human cells. Also, QDs can offer unprecedented interactions with the biomolecules on the surface of cells or inside the cells.^25^ Inorganic QDs have recently received much attention from the scientific fraternity for delivering anticancer drugs for the treatment of GBM. To cross the blood-brain barrier (BBB), QDs need to be of ultra-small size so that they can potentially pass through the tight junctions of the BBB more readily than larger molecules, enabling better penetration into the brain parenchyma.

 Also, QDs coated with specific molecules (ligands) can bind to receptors on the endothelial cells of the BBB. This triggers their internalization into the cells and allow them to pass through the BBB, to deliver the chemotherapeutics directly to the tumor site. These exceptional BBB penetration capabilities and the potential to target GBM tumor cells with low toxicity make QDs an ideal choice of nanocarriers for GBM therapy. Additionally, the higher accumulation of QDs in the specific tumor regions due to the enhanced permeability and retention (EPR) effect propose them as an effective drug delivery system against GBM.^26^

 The biocompatibility or toxicity concerns with the use of QDs can be addressed by i) coating the surface of QDs with materials like silica or polyethylene glycol, ii) conjugating QDs with specific antibodies that binds to specific GBM cancer cell receptors, minimizing the exposure to healthy cells, and iii) precisely controlling the size of the QDs affecting their cellular uptake and toxicity profiles, with smaller sizes often being less toxic.^27^

 Carbon nanodots, also known as carbon QDs (CQDs, primarily composed of carbon atoms, which are naturally found in the living body and are generally considered less toxic than heavy metals often used in traditional QDs), can reduce the toxicity of traditional QDs by offering a biocompatible alternative with a naturally occurring carbon structure. CQDs are less likely to trigger harmful cellular responses when introduced into the living body. Additionally, their surface chemistry can be easily modified to enhance water solubility and minimize potential interactions with biological systems, making them significantly less toxic compared to many other traditional QDs.^28^

 Use of toxic heavy metals *viz.* cadmium or lead poses additional risks to the health and therefore, creates major hurdles in scaling up QDs synthesis for human use. Additionally, the long-term stability concerns of QDs in biological fluids, complicated synthesis procedures, complex surface chemistry, immunogenicity, off-target effects of non-functionalized QDs, high costs of large-scale production, and low rates of reproducibility are few other challenges in translation of QDs into clinically applicable products on large scale.^29^

###  Glioblastoma (GBM)

 Cancer is a leading cause of death worldwide, accounting for 9.93 million deaths in 2020 and by 2040, the number of new cancer cases is expected to rise to 29.9 million. The brain and central nervous system (CNS) cancers (*viz.* tumors of the brain, spinal cord, meninges, cranial nerves, and spinal nerves) were recorded in 308,162 individuals, with a worldwide mortality of 251 329 individuals in 2020.^30^ GBM is classified into four subtypes *viz.* proneural, neural, classical, and mesenchymal. GBM, a grade IV astrocytoma, is a highly malignant brain tumor that has an assortment of chemotherapy-resistant, genetically unstable infiltrative cells.^31^ As there is no clear border between malignant GBM tumor cells and normal healthy cells, surgery alone is insufficient, and total resection is not feasible.^32^

 The best multimodal treatment involves radiation, chemotherapy, and surgery.^33,34^ GBM patients often have a poor prognosis, high death rates, and a median survival of 12 to 15 months, despite efforts to advance the therapy.^35,36^ GBM’s resistance to chemotherapy has hindered the development of effective treatment strategies.^37^ In furtherance of resistance problems, the BBB makes it difficult for systemic drugs to reach the tumor microenvironment (TME) in the brain, thus both challenges need to be addressed.^38^

 Patients undergoing cancer chemotherapy have considerable side effects because of the drug’s mode of action, which may impact the non-targeted healthy cells.^39^ Cytotoxic chemotherapy alters DNA and protein expressions even in normal host cells, resulting in a narrow therapeutic window and potentially fatal damage.^40,41^ Most chemotherapeutic drugs generally destroy the DNA or microtubules, thereby damaging the cells that can replicate quickly throughout the body.^42^ Common adverse effects of chemotherapeutic agents include anemia, tiredness, appetite loss, stomach and intestinal problems, myelosuppression, mucositis, alopecia, sterility, infertility, immunosuppression, and peripheral neuropathy.^30^

###  Monoclonal antibodies 

 The mAbs are utilized in GBM treatment due to their high specificity and affinity for biological targets, enhancing immunotherapy and antiangiogenic actions.^43^ The variable domain of a mAb is formed from the amino-terminal ends of an immune-globulin polypeptide and regulates its affinity for antigen binding.^44^ Antibodies, commonly known as immunoglobulins (Ig), are big, Y-shaped proteins that help the immune system recognize and eradicate the dangerous bacteria and viruses.^45^ These mAbs specifically attach to antigens on cancer cells, inducing an immune response against the target cancerous cells. Table 1 enlists the mAbs-based cancer therapies approved by the FDA till year 2020.^46^

**Table 1:** FDA-approved mAbs-based cancer therapies

**Name**	**Antigen**	**Format**	Indications(yearoffirstapproval)*
**Unconjugated antibodies**			
Atezolizumab	PD-L1	Humanized IgG1	Bladder, Non-small cell lung (2016), and Triple-negative breast (2019) cancers
Avelumab	PD-L1	Human IgG1	Urothelial Carcinoma (2017) and Merkel cell carcinoma (2017)
Bevacizumab	VEGF	Humanized IgG1	Colorectal (2004), Non-small cell lung (2006), Renal (2009), Glioblastoma (2009), and Ovarian (2018) Cancers
Cemiplimab	PD-1	Human IgG4	Cutaneous squamous-cell carcinoma (2018)
Cetuximab	EGFR	Chimeric IgG1	Colorectal cancer (2004) and head and neck squamous cell carcinoma (2006)
Daratumumab	CD38	Human IgG1	Multiple Myeloma (2015)
Dinutuximab	GD2	Chimeric IgG1	Neuroblastoma (2015)
Durvalumab	PD-L1	Human IgG1	Bladder Cancer (2017)
Elotuzumab	SLAMF7	Humanized IgG1	Multiple Myeloma (2015)
Ipilimumab	CTLA-4	Human IgG1	Melanoma (2011) and Renal cell carcinoma (2018)
Isatuximab	CD38	Chimeric IgG1	Multiple Myeloma (2020)
Mogamulizumab	CCR4	Humanized IgG1	Cutaneous T-cell lymphoma (2018)
Necitumumab	EGFR	Human IgG1	Non-small cell lung cancer (2015)
Nivolumab	PD-1	Human IgG4	Melanoma (2014), Lung (2015), and renal (2018) cancers
Obinutuzumab	CD20	Humanized IgG2	Chronic lymphocytic leukemia (2013)
Ofatumumab	CD20	Human IgG1	Chronic lymphocytic leukemia (2014)
Olaratumab	PDGFRα	Human IgG1	Sarcoma (2016)
Panitumumab	EGFR	Human IgG2	Colorectal Cancer (2006)
Pembrolizumab	PD-1	Humanized IgG4	Melanoma (2014), Various (2015-)
Pertuzumab	HER2	Humanized IgG1	Breast cancer (2012)
Ramucirumab	VEGFR2	Human IgG1	Gastric cancer (2014)
Rituximab	CD20	Chimeric IgG1	B-Cell Lymphoma (1997)
Trastuzumab	HER2	Humanized IgG1	Breast cancer (1998)
**Antibody-drug conjugates (ADCs)**			
Gemtuzumab ozogamicin	CD33	Humanized ADC	Acute myeloid leukemia (2000)
Brentuximab vedotin	CD30	Chimeric ADC	Hodgkin’s lymphoma and Anaplastic large-cell lymphoma (2011)
Trastuzumab emtansine	HER2	Humanized ADC	Breast cancer (2013)
Inotuzumab ozogamicin	CD22	Humanized ADC	Acute lymphoblastic leukemia (2017)
Polatuzumab vedotin	CD79B	Humanized ADC	B-Cell Lymphoma (2019)
Enfortumab vedotin	Nectin-4	Human ADC	Bladder cancer (2019)
Trastuzumab deruxtecan	CHER2	Humanized ADC	Breast cancer (2019)
Sacituzumab govitecan	CTROP2	Humanized ADC	Triple negative breast cancer (2020)
Moxetumomab pasudotox	CD22	Mouse ADC	Hairy-cell leukemia (2018)
Ibritumomab tiuxetan	CD20	Mouse IgG1-Y90 or In111	Non-Hodgkin’s lymphoma (2002)
Iodine (I^131^) tositumomab	CD20	Mouse IgG2-I131	Non-Hodgkin’s lymphoma (2003)
Blinatumomab	CD19, CD3	Mouse BiTE	Acute lymphoblastic leukemia (2014)

*Indications and year of first approval for each antibody were accessed using the FDA drug database. (https://www.accessdata.fda.gov/scripts/cder/daf/). Note: Adapted from Zahavi and Weiner,^47^ an open access article distributed under the terms and conditions of the Creative Commons Attribution (CC BY) license http://creativecommons.org/licenses/by/4.0/.

 The antitumor effects of mAbs are mediated through multiple pathways *viz.* surface antigen cross-linking, antibody-dependent cellular cytotoxicity, complement-mediated cytotoxicity, inhibition of essential activation signals for cell development, cytokine environment alteration, and promotion of an active antitumor immune response. Tumor antigens such as EGFR, CTLA4, CD20, CD30, CD52, erbB2, and VEGF have all been investigated for targeted drug delivery into the brain.^30,46,47^ Various antiangiogenic drugs have been utilized in GBM therapy to target the vascular endothelial growth factor (VEGF), reducing excessive vascularization of gliomas, and increasing tumor survival rates. These medications include macromolecules such as mAbs and small-molecules *viz.* kinase or integrin inhibitors. Bevacizumab is the first antiangiogenic drug to demonstrate promise in progression-free survival for GBM treatment, either alone or in conjunction with chemotherapy.^43^

 Currently, there is not a single review paper available discussing the facts on mAbs-anchored QDs, specifically targeting the GBM tumor cells. This review addresses a significant void in the literature to identify the key knowledge gaps and inform future research priorities to develop more effective strategies against GBM therapy. The aim of the current review is to present a concise compilation of the investigations on mAbs-conjugated QDs as anticancer systems against GBM treatment. The prime objective of this review is to discuss the major challenges associated with the existing treatment options for treating GBM and how the mAbs-conjugated QDs have demonstrated the improved therapeutic efficacy as targeted immunotherapy in the destruction of GBM tumor. The current clinical status of the mAbs in the GBM treatment has been presented at the end of this article, which would urge the researchers to explore them in conjugation with the QDs-based drug delivery systems.

Table 2 enumerates the various mAbs as targeting ligands and respective receptors for targeted delivery in GBM.

**Table 2:** A summary of various mAbs as targeting moieties and respective target receptors for targeted delivery in GBM treatment

**mAbs**	**Targeted receptor**	**Outcomes**	**References**
Nimotuzumab, Cetuximab, Anti-EGFR (GC1118), Panitumumab, Necitumumab	EGFR	High grade GBM can be successfully treated with or without chemotherapy and radiation treatment with Nimotuzumab. Combination therapy may reduce the resistance.	^48^
(P)RR-Ab	(P)RR	A monoclonal (P)RR-Ab efficiently suppressed gliomagenesis, cell proliferation, stemness, and migration indicating it to be a viable therapeutic approach.	^49^
IL-13Rα2	IL-13	Targeting IL-13Rα2 receptor could trigger cell death in GBM, offering a potential therapeutic strategy with few adverse effects.	^50^
Bevacizumab	VEGFR	Bevacizumab, either alone or in conjunction with chemotherapy, has demonstrated an improved overall survival in patients with recurrent GBM.	^51^
Pertuzumab, Trastuzumab	HER2	Increased trastuzumab levels in cerebrospinal fluid in impaired blood-brain barriers, supporting ongoing therapy in radiotherapy-treated brain metastases, enabling personalized treatment	^52^

Abbreviation: EGFR: Epidermal growth factor receptor, (P)RP: (Pro)renin receptor, IL-13: Interleukin 13, VEGFR: Vascular endothelial growth factor receptor, HER: Human epidermal growth factor receptor.

**Figure 2:**
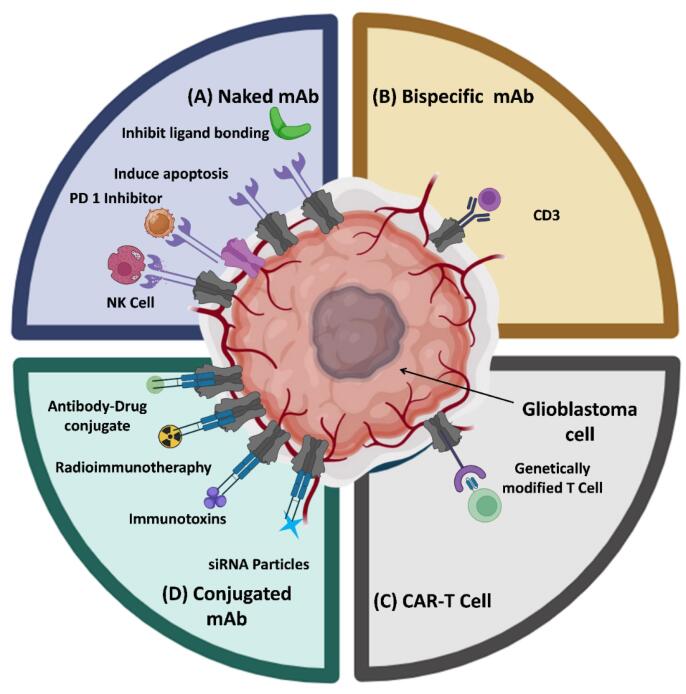
A schematic diagram presenting the potential of monoclonal antibodies in glioblastoma treatment

Figure 2 illustrates the various kinds of mAbs as immunotherapeutic agents for the treatment of GBM.

## Challenges in GBM treatment

 Inherent and adaptive heterogeneity, therapy-resistant stem cells, and highly developed metabolic machinery all contribute to the reduced glioma stem cells (GSCs) survival resulting in therapy failure. Clinical studies often failed because of insufficient concentrations in the brain due to presence of BBB and efflux transporters.^53^ GBM tumors are resistant to anticancer therapy due to their cellular heterogeneity, consisting of differentiated glioma cells, stem-like cells, and immune cells. GSCs contribute to therapy resistance not only by promoting the tumor heterogeneity, but also by modulating the components of the TME. GSCs are the most advanced lineage with stem cell-like regeneration capabilities, sharing markers with normal adult brain stem cells and progenitor cells. Recent single-cell RNA sequencing has revealed that transition of GBM cells from growth to differentiation phase, performs a key role in tumor development, treatment resistance, and recurrence.^54^

**Figure 3:**
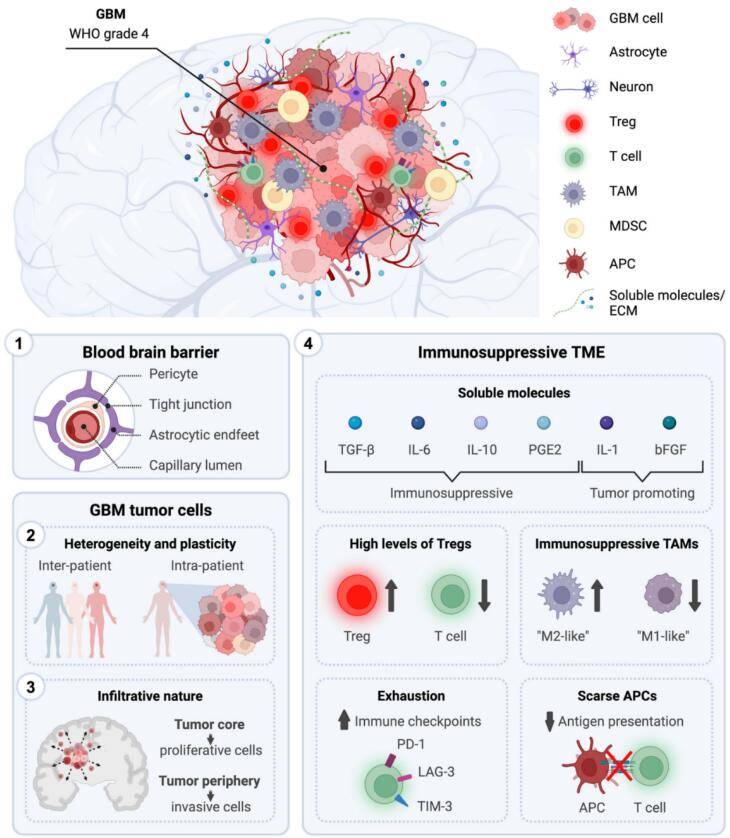
Therapeutic challenges for the cure of GBM. The figure illustrates the distinctive characteristics of GBM (WHO grade 4) that are understood to hinder the development of effective anti-tumor therapies. These includes: (1) an anatomical location shielded by the blood–brain barrier, (2) intra- and inter-patient tumor heterogeneity, (3) infiltrative behavior, and (4) a highly immunosuppressive TME. [Adapted from Salvato and Marchini,^55^ an article is licensed under a Creative Commons Attribution 4.0 International License, which permits use, sharing, adaptation, distribution, and reproduction in any medium or format]. Abbreviations: APC, antigen-presenting cell; bFGF, basic fibroblast growth factor; ECM, extracellular matrix; GBM, glioblastoma; IL, interleukin; LAG-3, lymphocyte-activation gene 3; MDSC, myeloid-derived suppressor cells; PD-1, programmed cell death protein 1; PGE2, prostaglandin E2; TAM, tumor-associated microglia and macrophages; TGF-β, transforming growth factor-β; TIM-3, T-cell immunoglobulin and mucin domain; TME, tumor microenvironment; Treg, regulatory T cell; WHO, the World Health Organization

 The development of effective treatments targeting GBM could plausibly be hampered by GBM’s unique traits, including its challenging anatomical location protected by the BBB, its invasiveness, the complexity of tumor variations within and between patients, and the immunosuppressive nature of the TME.Figure 3 illustrates the therapeutic challenges in the GBM therapy.

###  Blood-brain barrier 

 The BBB protects brain neural tissues and acts as a diffusion barrier, preventing toxins from entering the brain from the blood. It has two types of junctions: intercellular adherens junctions and paracellular tight junctions. An adult BBB consists of brain endothelial cells, basal membrane, pericytes, and end-feet.^56,57^ These intricately linked elements constitute a strong structure that considerably reduces the permeability of drug substances such as antitumor drugs.^58^ The brain capillaries are surrounded by astrocytes, pericytes, microglia, and neuronal processes, all of which are intimately related.^59^ In brain, the capillary endothelial cells have specialized barrier properties to ensure the homeostasis and protection of the CNS.^60^

 In addition, BBB emerges as a great challenge in delivering the mAbs-anchored QDs owing to low delivery rates (only a small percentage of mAbs administered peripherally can cross the BBB), disrupting the BBB for may lead to chronic neuropathological changes, and the low delivery rates contribute to the low success rate of GBM immunotherapy.^61^

###  Tumor microenvironment 

**Figure 4:**
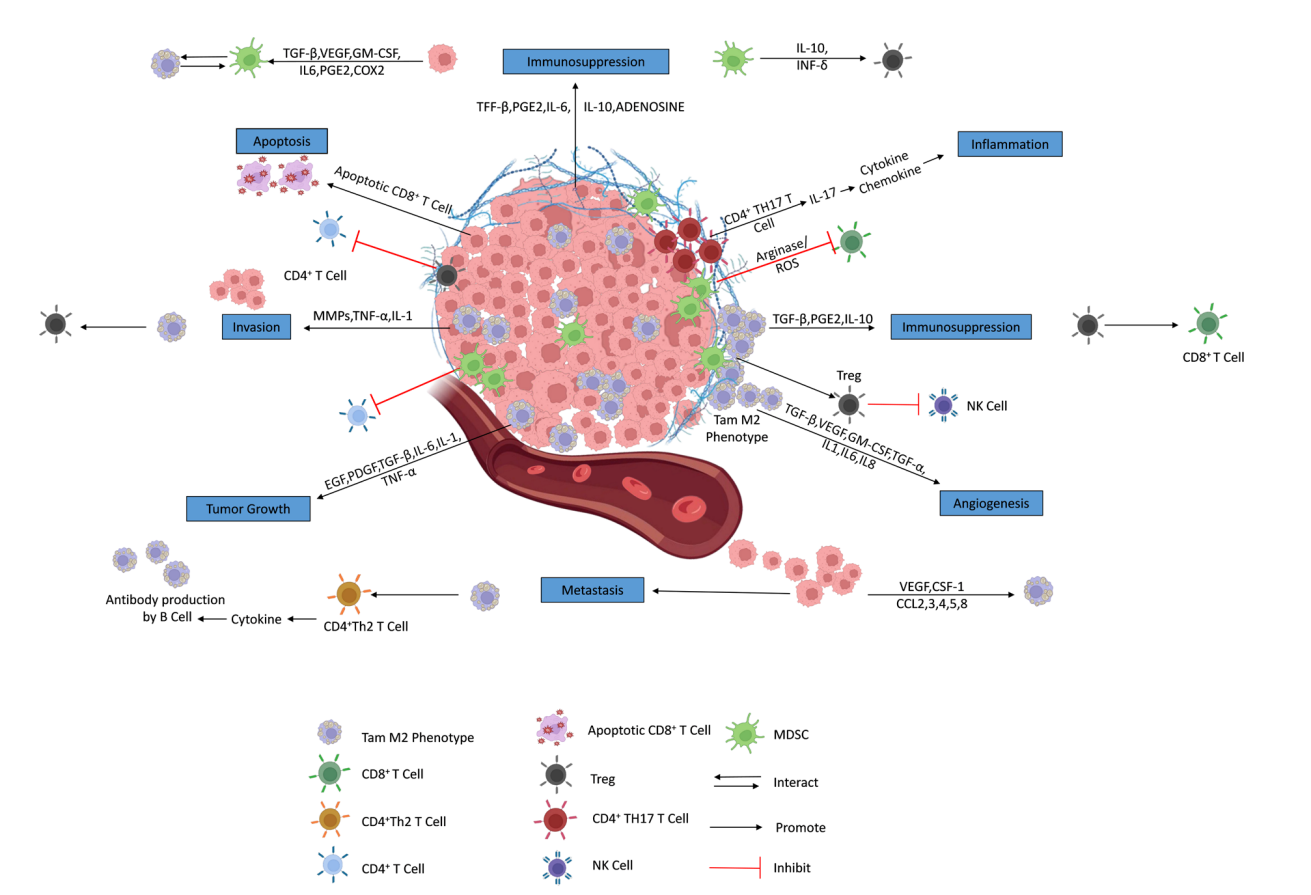
Immunosuppression mechanisms in tumor microenvironment. [Adapted from,^63^ an article is licensed under a Creative Commons Attribution 4.0 International License, which permits use, sharing, adaptation, distribution, and reproduction in any medium or format]. Abbreviations: TGF-*β*: transforming growth factor-*β*, VEGF: vascular endothelial growth factor, GM-CSF: granulocyte-macrophage colony-stimulating factor, IL: interleukin, PGE2: prostaglandin E2, COX: cyclooxygenase, INF: interferon, CD: cluster of differentiation, TH: T helper cells, MMP: matrix metalloproteinases, TNF: tumor necrosis factor, Treg: Regulatory T cells, TAM: tumor-associated macrophages, EGF: epidermal growth factor, PDGF: platelet-derived growth factor, CCL: chemokine (C-C motif) ligand, NK: natural killer cells, ROS: reactive oxygen species

 The invasiveness, development, and molecular heterogenicity of GBM are all greatly influenced by the interactions between tumoral cells and the TME.^62^ The perivascular niche, glioma cells, GSCs, immune cells, neuronal cells, communication factors, extracellular matrix, and chemical elements including pH and oxygen levels make up the TME of GBM. Numerous neoplastic and non-neoplastic cells, including macrophages, astrocytes, neuronal precursor cells, and microglia are part of the GBM TME. Understanding the functions of non-neoplastic cells, which account for 30% of tumor volume, can aid in the discovery of new targets for GBM treatment. Tumor-associated macrophages (TAMs) regulate tumors, whereas astrocytes support tumor development and preserve the integrity of the BBB. GBM TME, particularly its extracellular matrix (ECM), soluble factors, and growth factors, is crucial for cancer progression. The dynamic interaction between abnormal tumor cells, ECM, and immune system is essential. Figure 4 illustrates various immunosuppression mechanisms in GBM TME.^63^

 The efficiency of mAb-QDs systems for targeting the diverse GBM tumor cell populations has been demonstrated by several studies *viz.* anti-PD-L1 (aPD-L1) antibody-conjugated reduced graphene oxide QDs (rGOQDs) targeting the PD-L1 receptors,^64^ VEGF antibody conjugated Ag-In-S/ZnS QDs targeting the VEGF receptors,^65^ and anti-EGFRvIII-conjugated near-infrared QDs (Qd800) targeting the EGFR receptors on the GBM tumor cells,^66^ and so on.

###  Tumor heterogeneity

 Drug concentrations in the circulatory system are influenced by their metabolism and excretion in the human body, which in turn affects their capacity to enter the CNS.^67^ Drugs can interact with certain chemicals, which lowers their concentration and prevents their passage across the BBB, which is heterogeneous and can differ between various parts of the CNS.^68,69^

 One of the main causes of drug resistance and treatment failure is tumor heterogeneity, which is a characteristic of any cancer. Drug resistance affects the treatment targets and modifies the TME. Effect of tumor heterogeneity on drug resistance is shown by developments in molecular profiling methods.^70^ As intra-tumor heterogeneity refers to the different cell populations inside a tumor that show varied resistance to therapies, whereas inter-tumor heterogeneity refers to the variations in tumor types among patients.^71,72^

 The intra-tumoral heterogeneity significantly affects the efficacy of treatment in GBM, as the diverse cell populations within a single GBM tumor can port sensitivity to the therapy, often leading to treatment resistance and tumor recurrence, presenting one of the major hurdles in the efficient management of this highly aggressive brain cancer.^73^ Although there is no report available mentioning the potential of mAbs-conjugated QDs in overcoming the intra-tumoral heterogeneity, however combining different treatment modalities *viz.* surgery, radiation, chemotherapy, and targeted therapies to target diverse tumor cell populations may address some tumor heterogeneity issues in the GBM.

## Strategies to address the limitations of GBM therapy

 Despite safety issues, nanoparticles show great promise for advanced neurological therapy, could overcome the BBB constraints, and transform the treatment of CNS disorders.^74^ Larger biomolecules, that cannot cross the BBB owing to their size and polarity can pass across the BBB *via* receptor-mediated transcytosis, involving ligand interactions with specific receptors on the BBB.^75^ Ligand-conjugated QDs can cross the BBB to deliver drugs into GBM cells.^76^ Hanada et al^77^ investigated the QDs with varying sizes and surface charges in a BBB-transwell model.^77^ QDs of 2-10 nm size and selective qualities with surface modification allows them to diffuse in the brain by crossing the BBB. These QDs migrating over the BBB could provide both therapeutic effects and bioimaging, concurrently.^75^

 GBM, a low-prognosis cancer, has been treated with various therapies, including targeting tumor necrosis factor (TNF) to enhance the survival, well-being, and overall health of GBM patient. However, GBM’s immunosuppressive TME and drug resistance have hindered the success of anti-tumor therapy. Anti-VEGF drugs like bevacizumab and cetuximab have shown promising results in GBM treatments. However, the aggressive nature of GBM and a complicated TME structure makes the GBM therapy difficult.^43^ QDs could be delivered *via* invasive parenteral or noninvasive nasal routes to overcome the obstacles of TME in GBM.^78,79^

 Advantages of combinational drug therapy include low toxicity, overcoming potential drug resistance, and a synergistic effect. In contrast to the single drug delivery methods, which only have anticancer effect through one pathway, combination therapy functions through several pathways. Cells in brain tumors vary in their gene expressions and response to treatment, a phenomenon known as intra-tumor heterogeneity. A normal tumor subpopulation may be successfully eradicated by a single medication, but the resistant population will keep expanding. By focusing on several cell types, combinatorial treatment lowers the drug resistance while raising the rates of malignant tumor cell death.^80^ QDs produce tumor cytotoxicity *via* a variety of processes *viz.* oxidative stress, cell membrane destruction, DNA damage, cadmium ion release, cell surface adsorption, and modifications of cellular morphology. These impacts may harm nucleic acids, enzymes, and biological components,^81^ resulting in synergistic effects in GBM therapy.^82^ Surface modifications may further enhance the possibility of targeted delivery overcoming the chances of off-target toxicity.^83,84^

###  Monoclonal antibodies anchored QD delivery for GBM therapy 

 Intracranial administration of the chemotherapeutic agents could bypass the BBB, but it is inherently invasive form of treatment and mostly rely on diffusion of drug from the carrier into the bulk of the tumor. QDs, tiny nanoscale particles, can efficiently transport chemotherapeutics into targeted cells when combined with mAbs, which selectively targets specific cells or receptors.^85^ This may reduce adverse effects and improve treatment effectiveness. The mAbs coupled with QDs can attach to certain proteins on tumor cell surfaces, making them a valuable tool for cancer diagnosis and therapy by navigating tumor cells.^24^

 The way of conjugating the mAbs on the surface of QDs may affect their binding affinity, and therapeutic efficacy, as well. If the antibody is conjugated to the QD surface in a way that blocks its binding site, then the antibody would not be able to bind to its intended target receptor leading to poor therapeutic efficacy. Moreover, if the antibody is conjugated to the QD surface in a way that result in its premature release before reaching the target receptor, then the antibody may not be able to elicit its intended therapeutic activity at the target site.^86^ The mAbs could be conjugated on the surface of QDs using either a protein or a chemical linker. If imaging of the tumor cell is intended, then it is essential to use such a method that results in high antibody binding specificity. On the other hand, if therapeutic delivery to the tumor cell is intended, then it is essential to use such method that allow the releases of antibody from the QDs at the target site. Thus, the method of conjugating antibody on the surface of QDs is an important aspect that need to be considered as this may have a significant impact on the therapeutic efficacy.^87^

 While anchoring the antibody on the surface of the QDs, impacting their binding affinity and therapeutic efficacy, several factors need to be considered, which includes: i) orientation of the antibody, ii) number of antibodies per QD, iii) stability of the antibody-QD conjugates, and iv) release of antibody from the QDs.^86,87^

**Figure 5:**
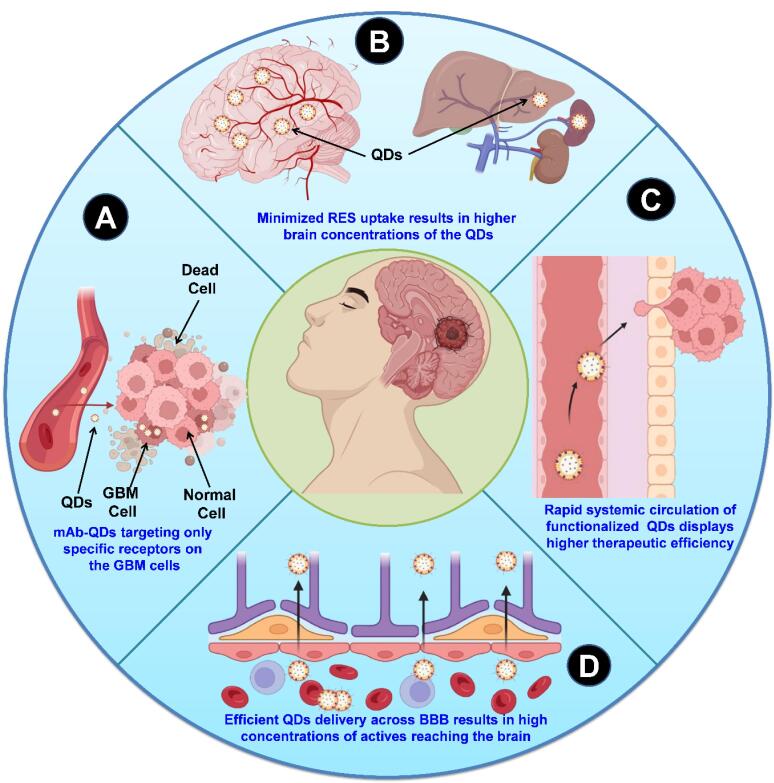
A schematic presentation of the mAbs-anchored QDs-based drug delivery strategies in overcoming barriers in the immunotherapy of GBM

 The mAb-anchored QDs showed promising potential as a delivery system against GBM and their efficiency is attributed to their prolonged blood circulation in the body (Figure 5A), target-specific delivery (Figure 5B), lower uptake by the reticular endothelial system (RES) (Figure 5C), and efficient transport across the BBB (Figure 5D),^85^ allowing more effective delivery of therapeutic payloads to the target GBM tumor cells.^88^

**Figure 6:**
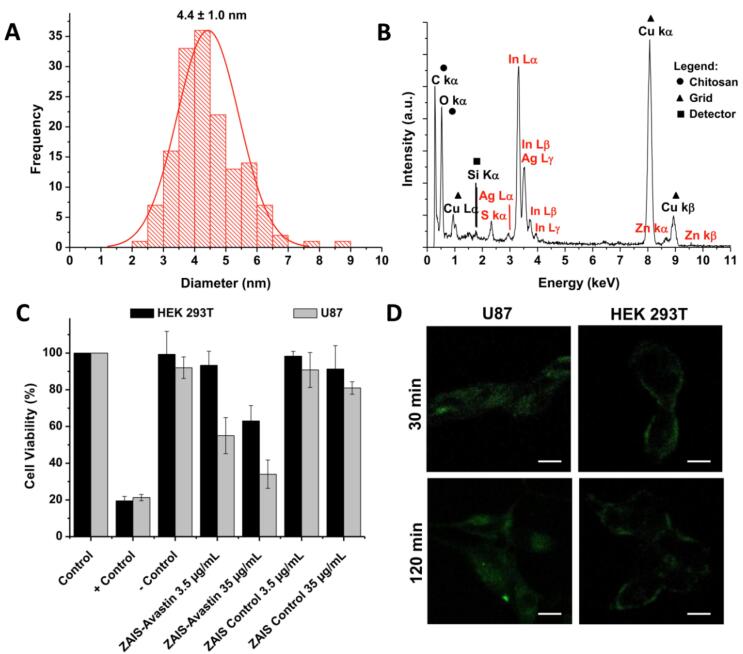
Size distribution, chemical composition, cell viability, and cellular uptake study of ZAIS/Chi (Ag-In-S/ZnS QDs stabilized by a chitosan polysaccharide and biofunctionalized with a mAb as targeting ligand). (A) Histogram of size distribution, and (B) EDX spectrum of ZAIS/Chi, (C) MTT cell viability results for U87 and HEK 293T cells incubated for 24 h with: ZAIS/Chi (control) and ZAIS/Chi-abVEGF, and (D) CLSM images (bright field + PL) after 30 and 120 min of contact with ZAIS/Chi-abVEGF. [Adapted from Santana et al^65^ with kind permission of the copyright holder, Elsevier, Amsterdam]

 Santana et al^65^ developed nanoconjugates containing Ag-In-S/ZnS QDs stabilized by a chitosan polysaccharide and biofunctionalized with a mAb targeting the VEGF receptors with intended GBM immunotherapy. These nano-immunoconjugates demonstrated bifunctional bioimaging and cytotoxicity against GBM cells. A facile one-pot aqueous synthesis method was used to synthesize the AgInS_2_ (AIS) nanoconjugates, where chitosan (Chi) was used as a stabilizer while silver, indium, and sulfide were used as salt precursors. The AIS nuclei acted as seeds for deposition of a ZnS layer, producing core-shell semiconductor nanostructures (ZnS-AgInS_2, _ZAIS), and finally coated with Chi as a capping ligand (ZAIS/Chi). The ZnS-AgInS_2_ nanostructures were coated with chitosan as capping ligand (ZAIS/Chi), and then bioconjugated with anti-VEGF mAbs (abVEGF, Avastin) to produce immunoconjugates. Transmission electron microscopy (TEM) and energy-dispersive X-ray spectroscopy (EDX) were used to evaluate the morphology and chemical properties of the nanoconjugates, respectively. The results indicated monodispersed spherical nanoparticles with an average size of 4.4 ± 1.0 nm (Figure 6A) contained Ag, In, Zn, and S as the main chemical components (Figure 6B).

 ZAIS/Chi-abVEGF QDs were tested for cytotoxicity on brain cancer cells (U87) and normal cells (HEK293T) using the MTT assay (Figure 6C). Zeta potential tests revealed that the amino groups of the cationic chitosan (R-NH^+^_3_) influenced the zeta potential values of ZAIS/Chi nanoconjugates at pH 6.5. Bioconjugation decreased the zeta potential values suggesting the formation of amide bonds between chitosan and mAbs. The hydrodynamic diameter of QDs in water at physiological pH was determined using the DLS (dynamic light scattering) technique. A study investigated the covalent bioconjugation of anti-angiogenic antibodies (abVEGF) with chitosan to produce fluorescent nanohybrids for cancer immunotherapy. Following bioconjugation, the nanoconjugates significantly reduced the cell viability response of glioma cancer cells (U87), with mortality of around 65% at higher doses. The nanohybrids were also examined for cell bioimaging, which revealed efficient internalization with green fluorescence under confocal laser scanning microscopy (CLSM) (Figure 6D). The investigation confirmed a unique nanotheranostic approach for targeting and abolishing the brain cancer cells *in vitro* utilizing anti-VEGF vectors.^65^

 Patel and Shah^89^ synthesized graphene QDs (GQDs) and functionalized them with Caspase-8 and trastuzumab using carbodiimide-amidation activation. GQDs were synthesized using two bottom-up approaches *viz.* hydrothermal, and pyrolysis. Particle size was measured using the DLS in three stages of formulation: diluted GQDs, purified and dialyzed GQDs, and raw synthesized GQDs. Without surface passivation, the purified GQDs was about 36 nm, significantly reduced to 5.2 nm after proper dilution in deionized water. Similarly, after

 purification of surface passivated GQD through PEG-6000, the particle size was increased near to 84 nm, which decreased to 56 nm after proper dilution. While the medium and scattering angle were corrected manually to monodisperse at 90°, the size of GQDs was significantly decreased to 27 nm. TEM scans revealed that GQDs had a quasi-spherical form and were evenly dispersed in an aqueous media. The lateral size of citric acid monohydrate (C.A.) QDs (CA-GQDs) was 6.36 nm before PEGylation, and it increased by approximately 24.10 nm after PEGylation. Cane sugar (C.S.) GQDs had a larger particle size (136.75 nm), which increased above 200 nm after PEGylation. AFM measured nanomaterials’ topographical appearance in orange subfractions. CA-GQD were well dispersed, with diameters around 6.85 nm and thickness 1.0-3.5 nm. After PEGylation, diameter increased by about 27.5 nm. The study characterized the conjugation of GQD-antibodies/proteins using FT-IR spectroscopy. The results showed that EDC/NHS amidation conjugation was stable and rapid compared to PEGylation.^89^

 GQD conjugates were exposed to *in-vitro* cytotoxicity studies in SK-N-SH (human neuroblastoma cell line) and N2a (a mouse neuroblastoma cell line) cell lines using MTT assay. The GQD conjugates have been investigated by fluorescence spectroscopy, FTIR, AFM, TEM, and DLS. The GQD conjugates exhibited modest acute toxicity in rat blood and dose-dependent toxicity in cell lines. Compared to other conjugates, the GQD_Caspase-8 conjugate exhibited superior anticancer and neuroprotective efficacy in the GBM tumor-bearing rat model. The effect of GQDs and its conjugates on SK-N-SH cell viability was investigated. Significant 50% cell mortality was seen after 6 h of incubation at a concentration 20 μg/mL of GQD and their conjugations, indicating a dose-dependent toxicity. After 6 h, the same GQD-conjugate concentrations demonstrated decreased cell mortality. After 24 h, however, fatal cell viability was noted at 50 μg/mL of simple GQDs. The MTT investigation assessed the effect of GQD and its conjugates on the cell viability of the N2a cell line. With a long log phase and moderate development, the N2a cell line displayed a typical growth curve. At greater doses, deadly cell death was seen, however GQD and its conjugates had negligible dosage toxicity. Following a 24 h incubation period, cell mortality was decreased by BSA (bovine serum albumin), Caspase-8, and trastuzumab conjugations. The findings indicated that brain tumor cells were protected by 50 μg/ml of GQDs.^89^

 In another study, rGOQDs have been used to develop a novel nanovehicle that can change the immune-boosting milieu of GBM. Lu et al^64^ targeted the PD-L1 on the surface of murine GBM cells. The nanoparticles loaded with the combination of immunomodulatory drug resiquimod (R848) and coupled with an anti-PD-L1 antibody (aPD-L1) can release R848 to improve the T-cell driven antitumor response.

**Figure 7:**
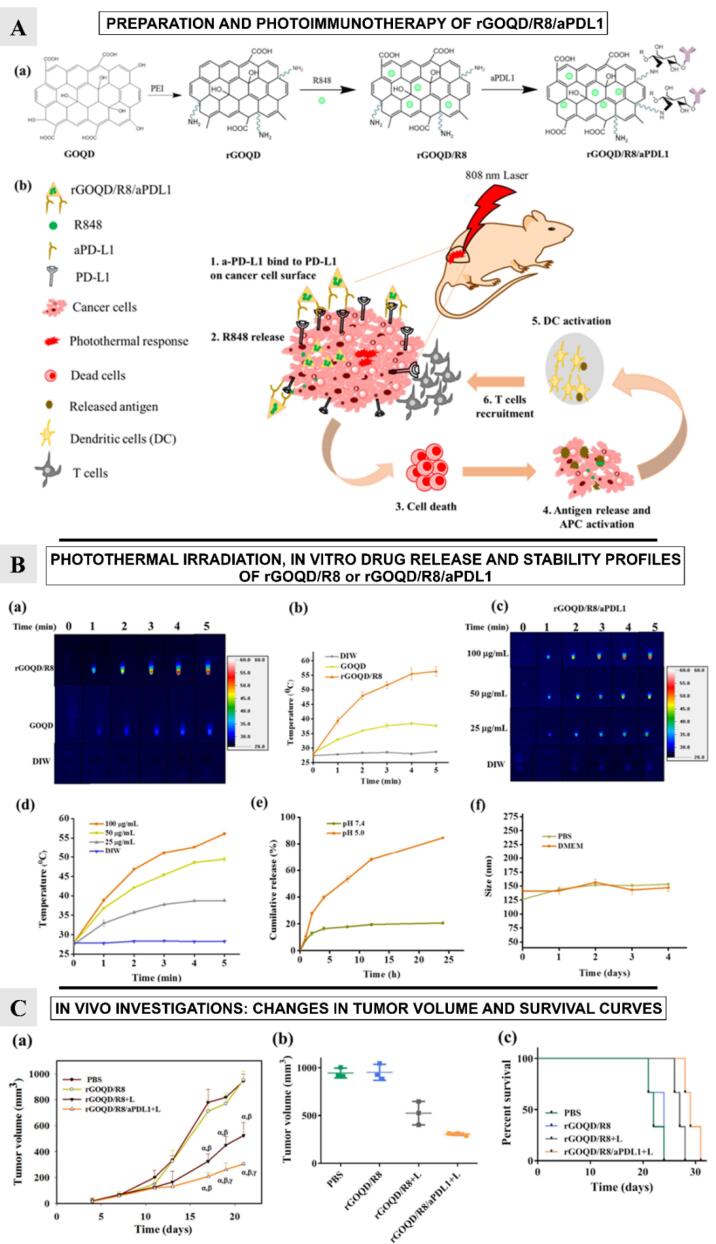
**[A]** Schematic illustration of the preparation of rGOQD/R8/aPDL1, (b) The photo-immunotherapy using rGOQD/R8/aPDL1 involves photothermal therapy and immune cell activation to exert an anti-tumor effect by (1) binding to the overexpressed PD-L1 receptors on tumor cell surface; (2) R484 release for activation of adaptive immune response; (3) photothermal-effect-induced cell death; (4) antigen release and antigen-presenting cells (APCs) activation; (5) dendritic cells (DCs) activation; (6) T cells recruitment. **[B]** The photothermal images (a), and the corresponding temperature profiles (b) by irradiating GOQD or rGOQD/R8 (100 µg/mL) with 808 nm laser (1.5 W/cm^2^) for 5 min. The control is deionized water. The thermal images (c), and the corresponding temperature profiles (d) by irradiating 25–100 µg/mL rGOQD/R8/aPDL1 with 808 nm laser (1.5 W/cm^2^) for 5 min. (e) The in vitro release of R848 from rGOQD/R8/aPDL1 at pH 5 and 7.4. (f) The stability of rGOQD/R8/aPDL1 in PBS and DMEM cell culture medium by measuring the particle size from DLS. All data are represented as mean ± SD (n = 3). **[C]** The *in vivo* therapeutic investigation: The tumor volume change (a), the scattered plot of tumor volume on day 21 (b), and the survival curve of animals (c) of ALTSC1 tumor-bearing mice after different treatments (mean ± SD, n = 3). The sacrificing criteria were when the tumor volume exceeded 1000 mm^3^. α *P* < 0.05 compared to PBS, β *P* < 0.05 compared to rGOQD/R8, γ *P* < 0.05 compared to rGOQD/R8 + L. [Adapted from Lu et al^64^ with kind permission of the copyright holder, Elsevier, Amsterdam]

 Utilizing a modified hydrothermal process, the rGOQDs were synthesized (Figure 7A-a). A flask containing 1% branched polyethyleneimime was filled with the GOQDs solution after it had been ultrasonically agitated for 4 h. The mixture was heated to 100°C before the rGOQDs (R848) was placed on top of it. After the separation of nanoparticles, the rGOQD/R8 pellets were again suspended in PBS. After adding activated aPD-L1 antibodies to the solution, the antibodies underwent purification. To measure the amount of rGOQD/R8/aPDL1, the Pierce^TM^ BCA protein assay kit was used. The rGOQD/R8/aPDL1 nanoparticles, with a size below 200 nm, showed 66% antibody conjugation efficiency, and their zeta potential changed from negative to positive after reduction with PEI, indicating successful drug loading.

 R848 and released damage-associated molecular patterns (DAMPs) together activate dendritic cells, enabling T cells to efficiently target and destroy PD-L1-suppressed glioma cells and promoting a strong photothermal immunotherapy (Figure 7A-b).

 The study explores the photothermal conversion capabilities of rGOQD/R8 and GOQD under 808 nm NIR laser irradiation. Results showed that the rGOQD/R8 increases temperature to 56 °C after 5 minutes, suggesting potential for cancer therapy. The temperature rise is concentration-dependent, with a peak temperature of 56 °C at 100 µg/mL nanoparticle concentration (Figure 7B-a-c). The study also tests the release profile of resiquimod (R848) from rGOQD/R8/aPDL1 under different pH values, indicating potential for release in acidic tumor microenvironments (Figure 7B-d-f).

**Figure 8:**
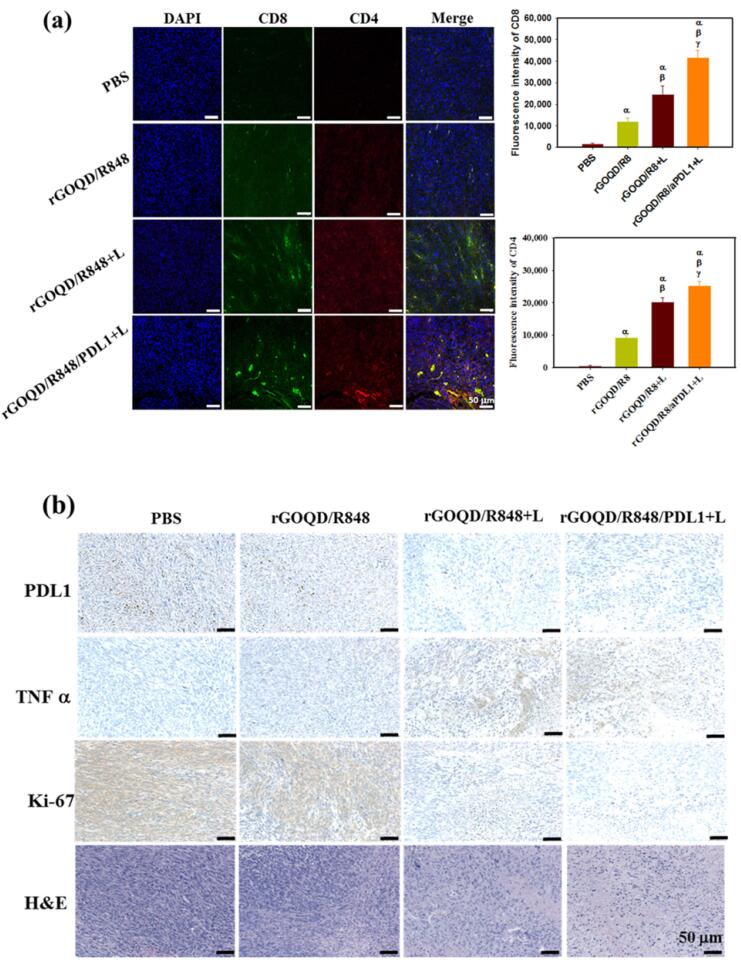
(a) The study examined the *in vivo* immune response to various treatments, measuring T cell infiltration into tumor tissues and comparing fluorescence intensity 19 days post-treatment. (b) The study analyzed tumor tissues 19 days after treatments, comparing PD-L1, TNF-α, and Ki-67 levels and H&E staining. Results showed significant differences compared to PBS and rGOQD/R8 + L. [Adapted from Lu et al^64^ with kind permission of the copyright holder, Elsevier, Amsterdam]

 Authors also studied the subcutaneous GBM tumor model and evaluated the change in tumor volume over a period of 21 days (Figure 7C-a-b). On 10th day, following first treatment, the tumor volume was found to be reduced for the rGOQD/R8 + L and rGOQD/R8/aPDL1 + L groups due to imminent photothermal-induced cell death. On 14th day, after second treatment, a slower tumor volume increase is noted for the rGOQD/R8/aPDL1 + L group compared to rGOQD/R8 + L group, due to the targeting toward the PD-L1 receptor on the cancer cell surface. After third treatment on 18th day, significant difference in tumor size starts to show between rGOQD/R8/aPDL1 + L and rGOQD/R8 + L groups. After the final 4th treatment, the significant difference in tumor volume still exists at day 21, with the mean tumor volume for the PBS group (944 mm^3^) being almost three times that of the rGOQD/R8/aPDL1 + L group (306 mm3). A survival curve of mice was constructed by setting 1000 mm3 tumor volume as the sacrificing criteria (Figure 7C-c). The nanoparticles showed nontoxic nature for 3T3 fibroblasts and ALTS1C1 GBM cells, with successful intracellular uptake over 24 h. Surface-conjugated aPD-L1 rGOQD enhanced the tumor targeting and intracellular uptake (Figure 8-a). Also, the immunohistochemical analysis demonstrated that the rGOQD/R8 + L treatments can significantly boost the expression of CD4 and CD8 in the tumor region for photothermal immunotherapy.^64^

 The study also assessed the biocompatibility of rGOQD/R8/aPDL1 nanoparticles using MTS assay to determine their potential toxicity toward 3T3 fibroblasts and ALTS1C1 cancer cells *in vivo*. Particularly, the rGOQD/R8/aPDL1 + L group exhibited an increase in TNF-α expression, substantiating the initiation of photo-immunotherapy(Figure 8-b).^64^

 A study on Cy5.5-tagged nanoparticles found that they can effectively treat subcutaneous GBM tumors in mice. The nanoparticles were injected through the tail vein of tumor-bearing mice, and fluorescence signals were detected for excised major organs and tumors. The study also found that both nanoparticles can activate the immune system through photothermal therapy (PTT), which is expected to express surface CRT (Calreticulin) protein on dying cancer cells. The combination of R848 and PTT can activate DCs (dendritic cells) and recruit T cells into the cancer region for combined photothermal immunotherapy. The investigation demonstrates that by inducing immune responses and improving the recruitment of cytotoxic T-lymphocytes to the tumor site, rGOQD/R8/aPDL1 + L therapy increases the immunogenicity of GBM cancer cells. For photothermal immunotherapy, the treatment increases the expression of CD4 and CD8 in the tumor location. It also increases the recruitment of CD4 + and CD8 + T cells to the tumor site, which increases T-cell infiltration. When paired with PTT, this treatment also improves survival rates in the mouse model. For a coordinated anti-tumor response, the study also emphasizes the possibility of combining a PD-L1 inhibition with R848 and PTT.^64^

 Papagiannaros et al^90^ prepared the tumor-targeted near infrared imaging agent composed of cancer-specific monoclonal anti-nucleosome antibody 2C5, coupled to QDs-containing polymeric micelles, prepared from a polyethylene glycol/phosphatidylethanolamine (PEG-PE) conjugate. Authors reported that the imaging potential of the targeted QDs-loaded PEG-PE micelles is 2-folds greater than the non-targeted QDs containing similar micelles.

**Figure 9:**
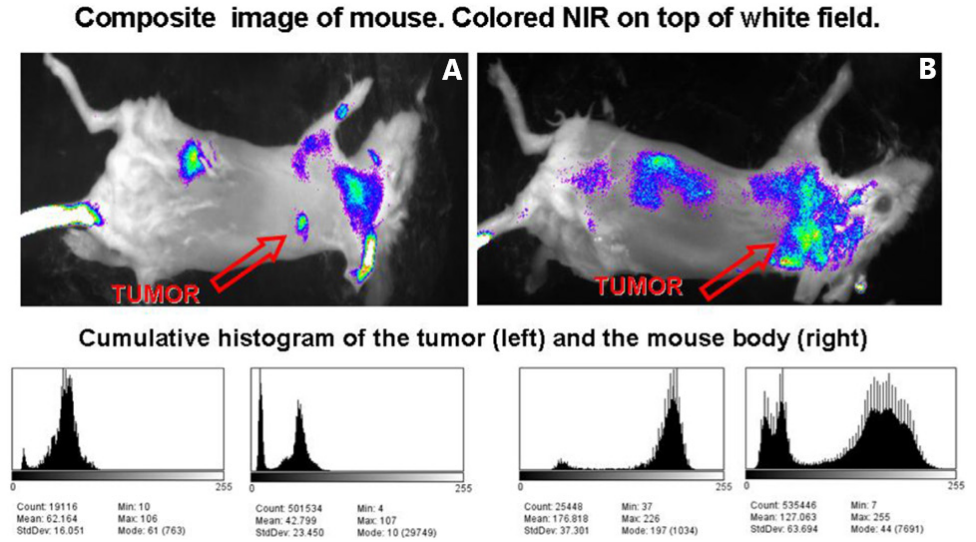
Composite images (white field image superimposed with the fluorescence intensity), and cumulative histograms for the tumor region and the whole body of two mice (A and B) injected with 2C5 QD-Mic. Fluorescence is concentrated mainly in the tumor area, and the cumulative histograms of the frequency of pixels *vs.* their value for the tumor area and the body of the animal verify that the region of interest (tumor area) has a narrow distribution of the highest value pixels from the animal body. [Adapted from Papagiannaros et al,^90^ an article licensed under a Creative Commons Attribution 4.0 International License, which permits use, sharing, adaptation, distribution, and reproduction in any medium or format]

Figure 9A & B (upside panel) illustrates the composite NIR pictures, superimposed over a white field image, of two mice injected with 2C5-QD-Mic 1 h after the injection. The signal, indicated by the arrow, is visible only from the tumor area. Some signal was detected from hairs that were not completely removed. The histograms of the pixel values (downside panel) verified this conclusion. Pixel values for the tumor area had the highest values compared to the rest of the animal body. For instance, the mean value in the non-tumor ROI is 42.8 ± 23.5, and 62.2 ± 16.1 in the tumor area. It is of particular interest that the pixel distribution is much narrower in the tumor ROI. The high slope of the pixel value distribution allowed the tumor to be identified clearly. The targeted QDs-loaded PEG-PE micelles produced ultrabright tumor images and doubled the fluorescence intensity compared to the passively targeted micelles, much rapidly and at the same low doses. This represents a concrete approach that may potentially serve to enhance early detection of tumor metastases including GBM.

Table 3 enlists the various investigations demonstrating the use of mAbs-anchored QDs in GBM treatment.

**Table 3:** Different monoclonal antibodies-anchored QDs for glioblastoma

**Monoclonal antibody**	**Quantum dots**	**Target receptor**	**Cell line**	**Preclinical Model**	**References**
Anti-PD-L1	GQD	PD-L1	ALTS1C1 cells	Mice	^64^
VEGF antibody	Ag-In-S/ZnS	VEGF	U87 and HEK 293T	-	^65^
anti-EGFRvIII	Qd800	EGFR	U87MG-EGFRvIII	Mice	^66^
Anti-EGFR	QD 525 streptavidin	EGFR	SKMG-3, U87	-	^91^
Trastuzumab and Caspase-8 antibody	GQD	HER2	SK-NSH and N2a	Rat	^89^
2C5 antibody	CdSe QDs	-	-	Female Balb/c mice	^90^

Abbreviations: Epidermal growth factor receptor: EGFR, PD-L1: programmed death ligand 1, VEGF: vascular endothelial growth factor, GQD: Graphene Oxide Quantum dots, HER2: human growth factor receptor 2.

## Clinical status

 A brief summary of various clinical trials on mAbs investigated for the GBM treatment are listed in Table 4. MEDI9447 and other mAbs have demonstrated potential in the treatment of GBM. In preclinical settings, it demonstrates promise by specifically inhibiting CD73 activity. Its safety, tolerability, and clinical efficacy are being evaluated in a phase I study.^92^ The mAbs have also been utilized to improve the immunotherapy and antiangiogenic processes in chemotherapy procedures.^47^ These advancements demonstrate continued initiatives to enhance patient outcomes and quality of life.

**Table 4:** A summary of clinical trials on mAbs investigated for GBM treatment

**mAbs**	**Study ID**	**Summary**	**Status**	**Phase**
Depatuxizumab Mafodotin (ABT-414)	NCT01800695	The study is assessing the safety and pharmacokinetics of ABT-414 in individuals with GBM.	Completed	I
Nimotuzumab, Temozolamide and Radiotheraphy	NCT03388372	The study aimed to evaluate the clinical benefits and safety of nimotuzumab in standard combined treatment for newly diagnosed glioblastoma patients.	Completed	I
Nivolumab	NCT02529072	Study implies Nivolumab with DC Vaccines for Recurrent Brain Tumors	Completed	I
EGFR(V)-EDV-Dox	NCT02766699	The Cerebral EDV study aims to assess the safety and tolerability of EGFR(V)-EDV-Dox, its immune response, and effectiveness in treating recurrent GBM.	Unknown	I
Cetuximab	NCT01238237	Trial for a super-selective intraarterial cerebral infusion, is being conducted for treating relapsed/refractory GBM and anaplastic astrocytoma.	Completed	I
Trastuzumab Deruxtecan (T-DXd)	NCT06058988	This study investigates the penetration of tumors with T-DXd and its potential effectiveness in treating brain cancers expressing the HER2 protein.	Recruiting	II
Tiragolumab and Atezolizumab	NCT06328036	The phase II trial evaluates the safety, side effects, and effectiveness of atezolizumab combined with tiragolumab versus atezolizumab alone in treating recurrent glioblastoma patients.	Not yet recruiting	II

http://www.clinicaltrials.gov.in/.

 Limited permeability across the BBB, GBM tumor heterogeneity, immunosuppressive microenvironment, and the invasive form of the GBM that generally develops resistance to the mAbs therapy are few of the major hurdles in clinical translation of the mAbs in the treatment of GBM. In addition, identifying the specific antigen on the GBM cell surface that can be targeted using mAbs is a challenging task that poses a major obstacle in the bringing the mAbs to the clinical settings for GBM treatments.^93^

 Developing engineered antibodies with improved BBB penetrability, novel targeted delivery systems that can directly deliver the antibodies to the target tumor site and combining the mAbs with multimodal treatment strategies *viz.* chemotherapy, radiation therapy, and use of immune checkpoint inhibitors may raise the chances of efficient translation of mAbs-based delivery for the effective management of GBM.^94^

## Conclusion

 Although advancements in the cure of GBM have evolved with prime objective of improving the overall survival rate of the GBM patients however, much remains to be done. Any treatment strategy should not only necessarily aim at reducing the size of the tumor, as recurrence and rapid proliferation of the tumor may eventually lead to patient’s mortality. Thus, the mAbs-conjugated QDs-based therapeutic regimen represents the improved targeted immunotherapy for safe destruction of the GBM tumor.

## Future perspectives

 The present review highlights the potential of surface-anchored mAbs-anchored QDs for the targeted treatment of GBM. The mAbs, with high target specificity and reduced toxicity to healthy cells, offer better relief over drug resistance which is much higher in chemotherapy and thus could be a promising option for treatment of deadly GBM. Herein, authors want to mention that the combination of mAbs with QDs could bring the synergy in the immunotherapy of GBM in combination with other therapeutic approaches *viz.* chemotherapy and radiotherapy. The development of novel approaches for scaling up QDs synthesis and improving the methods for conjugating mAbs on the QDs surface could be some future directions to be adopted by the researchers so that the clinically useful products could emerge with efficient potential in GBM therapy. In addition, researchers can explore the noninvasive intranasal route, which is yet to explore on large, for the delivery of mAbs-anchored QDs against GBM. The collective efforts focus on: i) deciding effective clinical trial strategies (selecting mAbs with proven safety, efficacy, and stability during preclinical assessment, mAbs with enhanced uptake and penetrability into GBM tumor cells) and ii) overcoming the regulatory hurdles (by developing safer, clinically more biocompatible, and therapeutically more efficient mAbs and QDs) for successful clinical translation of mAbs-anchored QDs based therapy for GBM treatment.

## Competing Interests

 Authors report no conflict of interest.

## Ethical Approval

 Not applicable.
